# The Examining Board and the Public Press

**Published:** 1893-09

**Authors:** 


### THE EXAMINING BOARD AND THE PUBLIC PRESS.

One of the weak arguments of those who opposed the examiners bill last winter was that it had not been asked for by the people; that it had been "sprung" upon the legislature by some Atlanta doctors, and that the masses of the people knew nothing of it. This is a fact, but a very poor argument.

The people and the members of the legislature should be taught what this bill is for, and those who are in favor of it can assist the good work by communications in their local paper. We would be pleased to see this matter thoroughly ventilated in the State press. Those who advocate this bill have nothing to fear from a free discussion, because he is "thrice armed that hath his quarrel just."

We have ourselves contributed a short article on this subject to the Atlanta *Constitution.* The friends of the bill throughout the State should see to it that this matter is brought home to the people through the public press. In this connection we were pleased to notice sometime ago an excellent article in the Montezuma *Record* from the pen of our lately deceased brother, Dr. R. O. Engram. We quote: "The interests of the people demand that men who are entering upon the practice of medicine should undergo a thorough examination and show competency before taking the health and life of people under their medical supervision. A medical examining board should stand between the people and the incompetent and fraudulent pretender. Our sister States about us have enacted laws requiring persons who intend to practice medicine to undergo examination, regardless of diploma; the result has been that the incompetent, the charlatan and the quack have found an asylum in the State of Georgia."

Several such articles throughout the State as the one from which we have quoted will accomplish a great deal toward the success of this bill in the fall. They would educate the people to the importance of this matter and help them to realize the fact that the establishment of a medical examining board is now a State's medical duty to its citizens.

---

WE have received the first copy of the *Journal of Surgery, Gynecology and Obstetrics*, published in this city and edited by Dr. C. Evans Johnson. The contents are well chosen and the issue is very creditable. "Collaborators" have been selected from both hemispheres. Among them are the names of several prominent physicians of this city, who are also associated with the other two journals of Atlanta. Our esteemed friend, *The Alabama Medical and Surgical Age*, sees in this latter fact a suggestion of consolidation, but "would be surprised to see this new journal absorb the two old reliables in Atlanta." The suspicions of our pink contemporary from Anniston are entirely without foundation. The 'two old reliables," that have reached a vigorous age, are not to be swallowed by a newcomer that has cut only one tooth.

We will add, however, that the *Journal of Surgery, Gynecology and Obstetrics* has our best wishes for a long career of success.

---

THE first quarterly report of the Atlanta Polyclinic shows that there have been over one thousand and one hundred cases admitted and over three thousand and five hundred treatments. There have been more than one hundred operations of various characters, including removal of the superior maxilla, celiotomy for extrauterine pregnancy, etc. The report is very gratifying and the condition of the institution highly encouraging.

